# The Impact of COVID-19 on People with a Visual Impairment in Northern Ireland: A Sensory Support View

**DOI:** 10.3390/ijerph21121701

**Published:** 2024-12-20

**Authors:** Laura N. Cushley, Matthew Mo, Tunde Peto, A. Jonathan Jackson

**Affiliations:** 1 Centre for Public Health, Queen’s University Belfast, Belfast BT12 6BA, UK; t.peto@qub.ac.uk; 2 School of Medicine, Dentistry and Biomedical Sciences, Queen’s University Belfast, Belfast BT7 1NN, UK; matthewmo32@gmail.com; 3 Department of Ophthalmology, Belfast Health and Social Care Trust, Belfast BT12 6BA, UK; jonathan.jackson@belfasttrust.hscni.net; 4 Northern Ireland Clinical Research Network, Belfast Health and Social Care Trust, Belfast BT12 6BA, UK

**Keywords:** sensory support, blindness, visual impairment, COVID-19, sight loss, sight impairment, impact

## Abstract

**Background:** The COVID-19 pandemic brought many challenges for all and especially for people with a visual impairment. As a result, many healthcare services had to close or be reduced, and new rules and regulations were implemented. These rules, regulations and testing procedures were challenging for many people with a visual impairment. **Methods:** Focus groups were conducted with sensory support workers at a regional sensory meeting in Northern Ireland. A set of semi-structured questions were asked about how services were conducted, the challenges faced by people with a visual impairment and any specific cases which showed its impact. Two researchers transcribed and analysed the focus group data. **Results:** The analysis resulted in four themes, namely “as a result”; healthcare; rules, regulation and testing; and mental and physical health. Some of the biggest issues mentioned were access to healthcare, COVID testing, online working, online schools, one-way systems, social distancing and the impact on mental and physical health. **Conclusions:** As expected, COVID-19 impacted people with a visual impairment. This study shows some of the barriers faced by people with a visual impairment, especially with regards to the rules and regulations. People with a visual impairment also found it difficult to access the necessary healthcare and support, or they were too fearful to seek it out. This paper provides an insight into the barriers faced by people with a visual impairment and how we may support them in the future.

## 1. Introduction

There are an estimated 57,500 people in Northern Ireland with a visual impairment, which is expected to rise by over 25 percent by 2032 [1]. The COVID-19 pandemic brought many challenges for the general population but especially for those who are more vulnerable, including people with disabilities. This sudden disruption to daily living caused by COVID-19 was exacerbated in people with a visual impairment, impacting their socio-economic status and livelihoods [2]. People with a visual impairment often had higher levels of loneliness [3] and difficulty adhering to the prevention measures [4,5]. There were also often issues with them receiving incomplete news due to word of mouth or gaining relevant information in an accessible format [4,5]. These accessibility issues were particularly relevant to those who used technology for work or school [6]. People with a visual impairment cited issues with getting their shopping done [5,7] and requiring more assistance than usual [5,7].

The COVID-19 pandemic also led to many hospital services being halted or reduced in capacity. Restricting access to services was shown to cause significant fear for people with visual impairments [8], and many were unhappy with the healthcare and social care they received during the pandemic [9]. In Northern Ireland, the number of people certified to have a visual impairment reduced drastically between March and June of 2020 [10]. This meant that people who may have needed services such as sensory support were unlikely to be registered during this time. Sensory support workers support and work with people with sensory impairments such as visual impairments and hearing impairments. Prior to the pandemic restrictions, sensory support workers would conduct an initial phone call with people with a visual impairment before conducting in-person visits. At in-home visits, sensory support staff would normally have provided ongoing sensory-based support, providing technical equipment and rehabilitation. This in-person support was, however, restricted, although support continued to be provided, primarily over the telephone. 

In addition, individual assessments of needs were much more difficult given the inability to assess how a person was completing their daily living tasks; access individuals’ living environments; or provide assistive technology and devices, appliances and mobility and orientation training, which could not be provided in person. Assistive devices such as magnifiers, raised dot stickers and liquid level indicators could be sent out but not installed, nor could usability instructions be given, by sensory support staff. Orientation and mobility training, using assistive devices such as canes, could also not be provided. 

This paper sets out the opinions and experiences from sensory support workers on the impact of COVID-19 on people with visual impairments.

## 2. Methods

Sensory support workers provide a key component of rehabilitation care for people certified to have a visual impairment. They provide ongoing social support throughout the diagnosis and life of people living with a visual impairment. The opinions and experiences of people working with visually impaired people closely in the community are invaluable. 

Focus groups were conducted as part of a round-table discussion at a regional sensory support training meeting in Belfast, Northern Ireland, in November 2022. There were 7 focus groups, with each comprising between 5 and 6 people. Each focus group included a variety of individuals who regularly worked with visually impaired patients, including support workers, charity leads and other healthcare professionals. The discussion took place in the visual impairment support section of the day, and it was made clear at the beginning of the discussion that it applied specifically to people with a visual impairment. It was noted that some of these people may have had dual sensory loss; however, this was not discussed explicitly. Throughout the event, only one case with dual sensory loss and a cochlear implant was mentioned. 

The questions, listed below, were distributed to each focus group, with the lead (J.J.) prompting each group on when to discuss each question. 

“We want to explore 6 areas where we feel your experience may help us understand more clearly how the COVID-19 pandemic impacted people with a visual impairment. Please answer these questions only in regard to visually impaired individuals (although we understand some have dual sensory loss). Please provide as much detail as possible without disclosing patient identifiable information.

1. COVID-19 and Social Isolation—Do you think COVID-19 impacted on VI and Certified patients more than normally sighted individuals. If so how?
2. COVID-19 and health care—Do you think COVID-19 impacted on patients access to health care support in both primary and secondary care. If so how?
3. COVID-19 and virus avoidance strategies—Do you think COVID-19 impacted on patients access to social care support both at a community level and secondary care level? If so, how?
4. COVID-19 and self-testing—Are you aware of any difficulties VI individuals has with undertaking COVID-19 self-testing to determine their infective status?
5. COVID-19 and technology—Did the impact of COVID-19 affect your patients ability and willingness to acquire or utilise new technology for communication or leisure?
6. Finally—Are there any other aspects of COVID-19, and its impact on VI people, that we haven’t already discussed’’.

J.J. is a senior male clinical academic in ophthalmology and holds clinical qualifications. J.J. works closely with sensory support workers to provide ongoing care and support to the visually impaired community. The discussions in the focus groups lasted around 40 min, with feedback and the overall discussion transpiring over 20 min periods. 

Scribes were present at each table, alongside audio recordings being taken—due to the number of people in the room, the audio recordings in each individual group were poor. Therefore, each focus group nominated a scribe and wrote down pertinent information. Each group was then asked to give feedback to the lead (J.J.). This feedback was also audio-recorded and scribed at the time. Audio recordings were used by the transcribers to ensure the accuracy and validity of the transcripts. 

After the final transcription, the transcription documents were imported into NVivo (Version 8, QSR International, Burlington, MA, USA) software for a qualitative analysis. This was conducted by two researchers (one experienced researcher (L.N.C.) and one junior researcher (M.M.)). Initial coding was undertaken to assess the data saturation and begin the thematic analysis [11]. Both researchers (L.N.C. and M.M.) then held a meeting to discuss common themes and decide on the final themes and subthemes to present to the senior researcher (J.J.) Any conflicts were resolved through a discussion between all of the researchers to come to a common decision. On discussion, all of the themes were finalised with no conflict. The names of the overarching themes were decided on by all 3 researchers in correspondence with the original questions and thematic codes. 

Caldicott Guardian approval for the project was given through the Belfast Health and Social Care Trust (BHSCT), ref 6301, and consent was given by all of the participants. 

## 3. Results

**Figure 1:**
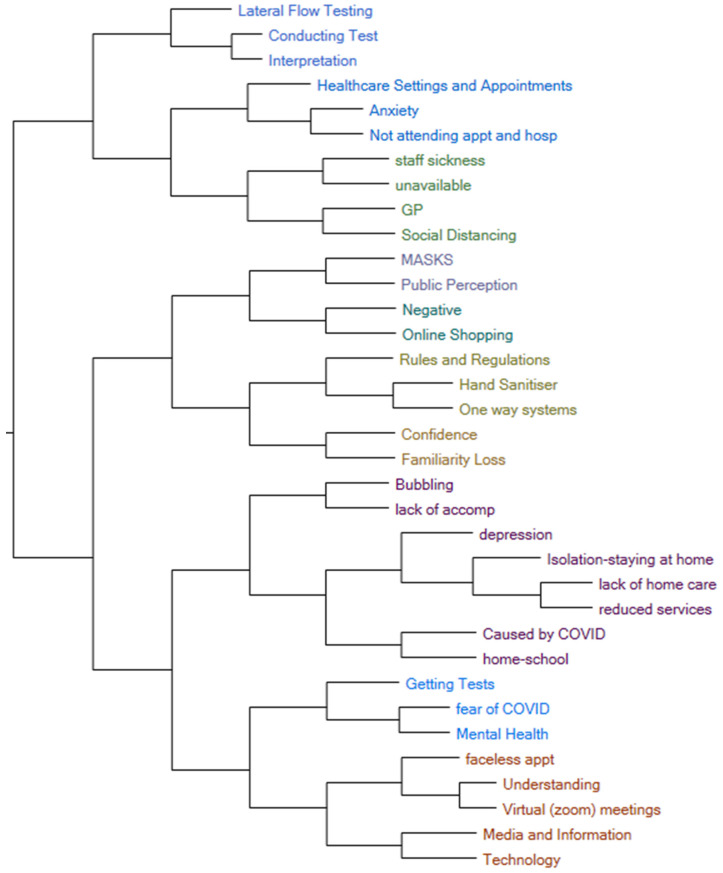
Network diagram of codes. Colours correlate with the different themes and subthemes they are assigned to.

On analysis of the focus group notes and transcripts, the themes, codes and subcodes (Table 1) were decided upon by the two analysts (L.N.C. and M.M.) and the senior researcher (J.J.). Figure 1 shows the network of codes assigned throughout the transcripts. 

### 3.1. As a Result

COVID-19 resulted in a loss of familiarity for everyone but especially for people with a visual impairment. The sensory support workers reported that “mobility requires familiarity, and this was gone”. This was further exacerbated by the fact that “patients couldn’t adhere to rapidly changing guidelines, and they were not accessible”. With “so many changes rapidly, people stayed at home”. There was an inherent and apparent loss of orientation, which is fundamental for independence. 

Alongside a loss of familiarity came a switch to reliance on technology for communication, learning and employment. The sensory support workers reported that many people did not have access to adequate technology or Wi-Fi pre-COVID-19. This was particularly a problem experienced by older patients living in remote rural areas. While “some people embraced tech … in [their] opinion the majority rejected any offer of support”. Generally, “older people feared technology, were inexperienced or were disinterested”. They reported that the telephone healthcare consultations and emotional support given by sight loss charities “simply didn’t work”. 

Difficulty accessing the appropriate technology was also noted to have an impact on visually impaired parents and carers who assisted with a child’s schoolwork. Many felt that their clients were not provided with adequate support by schools in using technology or assisting their children with the use of technology. Alongside this came an increase in the use of teleconferencing, including using Zoom, which was “not accessible at all”.

While there were many negatives associated with the unavailability and accessibility of technology, the sensory support workers stated that “COVID-19 did make technology better” and made reference to a “cochlear implant now [being able to be] linked to an iPad”. 

### 3.2. Healthcare

As a result of service restrictions, many healthcare appointments were delayed or cancelled. These delays and cancellations in some cases were caused by changes to facility access guidelines and waiting area restrictions, whereas in others, service reductions were caused by staff shortages and staff working from home. Low-vision and ophthalmology services were also impacted, as “all low-vision clinics were stopped” with “equipment being posted out”. This created issues with patients’ ability to use this equipment confidently and effectively. The way that people were registered as sight-impaired was also affected, with “certifications of visual impairment posted out, some got copies, some sent originals”. Some patients did not attend appointments due to a lack of access to healthcare or transport or fear of COVID-19. People with visual impairments also stated that due to reduced services at the Macular Clinic in Belfast, their injections were often delayed, causing worry and anxiety. Further worry and stress was caused as people had to “[attend] appointments without a family member present [which] increased anxiety”. 

Fear around contracting COVID-19 was reported to be a significant issue, with patients “so terrified of going to hospital in case they get COVID. Even people who were very seriously ill would not go to hospital”. The sensory support workers reported that one person with a severe sight impairment refused to go to hospital due to her fear, despite being very ill. The sensory support team “eventually had to convince them to go and get [them] an ambulance”. 

“Different Trust sensory teams developed different ways of working—telephone appointments were very different and no home visits (PHA)”. Many of the sensory support workers felt that “it was a disservice to patients” and “patients played down their issues, and it was an injustice to them”. Tele-appointments through phone or video calls were often conducted in place of face-to-face appointments. These were found to be extremely difficult for people with hearing impairments or other barriers to communication. There was also an inability to see and examine patients, and parents often found it difficult to accept a new diagnosis over the phone, so this was especially difficult for the paediatric population. 

In addition, there were barriers to receiving primary care, including from general practitioners (GPs). Difficulty in accessing information and contacting their GPs was reported by many clients. Even when they could contact their GPs, these appointments were often faceless. In fact, one group reported that they received a delayed referral involving serious self-harm (eye-poking), something which was not identified on time due to a lack of visual face-to-face contact. 

Due to staff unavailability and restrictions, rotas changed, meaning call times changed, and there was a reduction in available carers. Reduced services also included daycare centre closures or reduced hours and a reduced level of support that sensory services could offer. There was also a lack of home care; many people had to “go into care due to lack of physical services” despite having hospital rehabilitation through physiotherapists. This often caused clients to “lose skills, become institutionalised and lose confidence”. In addition, some people were discharged home due to a lack of availability of domiciliary care, which many felt often caused their sensory-impaired clients to experience a “lack of independence” and a loss of confidence. 

### 3.3. Rules, Regulations and Testing

As the pandemic progressed, many rules and regulations were brought in to protect vulnerable people, but these were particularly difficult for people with a visual impairment to adhere to. The sensory support workers reported that people with visual impairments stated that “everyone looks the same” with masks on and that they found it difficult to “identify people and visitors”. People with a visual impairment reported that masks were difficult to locate and awkward to wear. Spectacle wearers found that their lenses steamed up, impeding their vision, when they wore their glasses in conjunction with a face mask. Masks introduced a further barrier to communication, especially for people with a hearing impairment, as voices were muffled and they could no longer lip-read. 

People with a visual impairment often found it difficult to judge social distance and could not detect or read social distancing signs. Social distancing was also particularly difficult for cane users. It was reported that going shopping was increasingly difficult due to social distancing and one-way systems. One-way systems often kept changing, especially in primary care and shops. In addition, hand sanitisation on entering buildings was difficult for people with a visual impairment. The participants reported that clients could not locate dispensers or found it difficult to know how they worked. 

The sensory support workers reported that there was also a general “lack of transport services”, isolating people further. In addition, “bus drivers were very strict and anyone who wasn’t wearing a mask you get kicked off”. One worker reported that they were “on bus with a patient and the patient couldn’t understand description of journey due to mask but the sensory worker was able to show the info on phone/by type texting”.

In addition to these rules and regulations came restrictions on movement and visiting family and friends. “Bubbling” was introduced to allow people to expand their social interaction with close family and friends but was restricted to one other household. This could mean that only one person visited, and this caused isolation. The sensory support workers reported that clients were often “alone listening to the news—anxious and worried”. Sensory support workers also reported that clients were “very often dependent upon others for outings, creating further isolation”. 

**Figure 2:**
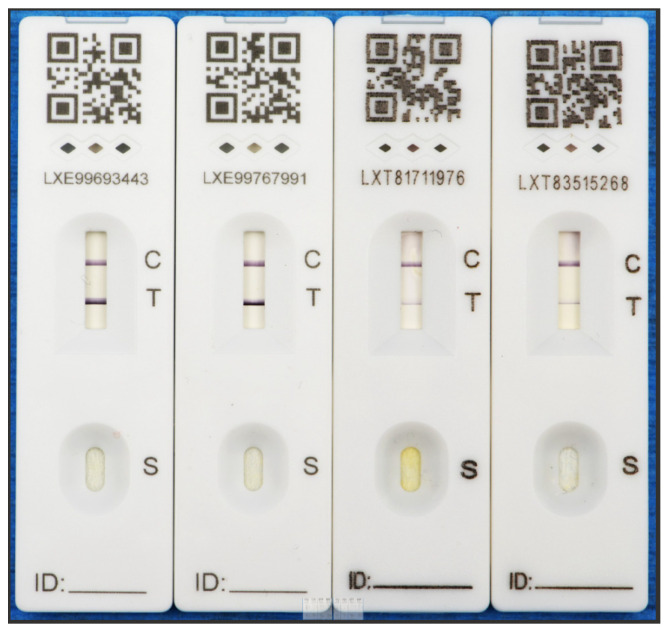
Example of 4 different positive COVID tests recorded at different stages of recovery, illustrating how low-contrast positive results can be very difficult to interpret in cases of vision impairments.

Figure 2 shows the issues with size of the tests and contrast of reading the lines throughout different stages of recovery. COVID-19 testing was “virtually impossible to use if someone has low vision”. The sensory support workers stated that “for someone with sight impairment or severe sight impairment—seriously disadvantaged—didn’t know how to self-test”. People with a visual impairment also “didn’t know how to get tests—missed info on TV, couldn’t do a test, couldn’t read test—especially if they lived alone”. The support workers reported that visually impaired clients stated that “even opening the packet was a challenge” and that “dropping the drop on the test was problematical”. Even after completing the challenge of conducting the test, people with a visual impairment struggled with its interpretation. Clients reported that they could not see the lateral flow and that it was very hard to “see the red lines”. This often meant that carers or sensory support workers had to help clients to complete tests, which put them at risk. 

As mentioned above, the information from COVID-19 packs, signs on regulations and information on COVID-19 in general were often inaccessible. People with a visual impairment reported not being able to see the information provided by post or on the television. Some also reported that the media often “scare-mongered”, and some visually impaired people felt they were in the increased-vulnerability group. 

### 3.4. Mental and Physical Health

Fear of COVID-19 transmission often caused the public to negatively react to a person with a visual impairment accidentally getting too close to them. People with a visual impairment often reported bumping into people, with negative reactions. Participants felt that the public had a poor awareness of visual impairment and the “struggles of people with a visual impairment”. In addition, the public perception of people with visual impairments not wearing masks or struggling to follow one-way systems was negative. 

All of these different aspects caused people with a visual impairment to have increased anxiety and reduced confidence. People were often scared to go out, except to attend medical appointments. In fact, one person who was ill and needed hospital attention would not go to the hospital due to fear of COVID and had to be convinced to call an ambulance. People cancelled on their carers due to fear of contracting COVID from visitors who had multiple forms of contact with others; this resulted in further isolation, which, in turn, impacted their physical health. There were also other barriers to going out, as transport services were diminished and reduced. Reports also suggest that transport workers were very strict about mask regulations and “anyone not wearing a mask was thrown off”. In addition, people with hearing loss could not communicate over the telephone, thus isolating them further. 

The sensory support workers also reported lasting impacts from COVID-19, including the isolation, mental health concerns and anxiety. The sensory support workers stated that they are “still meeting people who have lost confidence and only go out for medical appointments”. In addition, support groups and other social groups are still not meeting face to face, with sensory support workers feeling COVID-19 has “given people a rationale why we don’t come together, groups ceased and never returned, COVID used as the reason”. Below in Table 2 are some quotes from the focus groups. 

## 4. Discussion

COVID-19 had a profound global impact on those delivering, or those in receipt of, healthcare and social care support, creating barriers and enablers throughout. Some of the service changes, such as introducing telephone consultations and the triaging of appointments, have lasted long after the pandemic. Increased use of technology and video conferencing, which has demonstrated benefits for both sighted and non-sighted individuals when applied appropriately, have now become a component of routine care. This highlights the importance of tailoring the appointment types and delivery to patients’ sensory and other requirements. For example, telephone or video appointments might be very useful for someone who requires frequent appointments and cannot get time off education or employment but may not be helpful for a more elderly person or someone with sensory needs. 

Necessary precautions were essential during the pandemic to reduce viral transmission, hospital admissions, sickness and death. Despite these necessary precautions, they introduced further barriers for people with sensory impairments. The results from this study mirror those from the literature—that masks caused voices to be muffled and made facial expressions difficult to read and lip-reading impossible [9]. People with sensory impairments found mask-wearing difficult. In fact, many people with a visual impairment requested mask exemption certificates due to a mask further impeding their visual field. At the local context, many people with a visual impairment require exemptions from mask-wearing due to the further impairment of their visual field. All of this isolation, mobility restrictions and reduced exercise have negatively impacted people in general but especially people with a visual impairment. Our research shows one of the biggest barriers was hand sanitisation when entering buildings. The sanitiser stands were difficult to see and thus use and could also be trip hazards. In addition, due to the varying designs of these hand sanitisers, including foot pedals, people with visual impairment found them difficult to use. The need for hand sanitisation and the risk of infections likely further impacted people using tactile aids, such as braille [6,12], tactile maps and tactile cones under traffic lights. 

Our research echoes the literature, as social distancing and one-way systems became the new normal. Adhering to a 2-metre social distance was difficult, as judging distances was challenging [13,14]. Reports suggest that people with a visual impairment often bumped into other people or were closer than they should have been, with negative reactions from the public. People with vision aids such as canes and guide dogs [15,16] found this challenging. One-way systems were also reported to be challenging due to an inability to follow these systems or read the signs advising on how to follow them [8]. Despite this, some people with a visual impairment found the raised lines on the ground in one-way systems helpful for navigating obstacles. 

Due to social distancing, healthcare and other services were postponed or reduced. Our results and other reports suggest that low-vision services ceased, and other services ceased or were very reduced [2]. Due to the lack of face-to-face appointments, patients and parents were informed of life-changing diagnoses over the phone, with limited options for emotional support and explanations at the time. In our study, the sensory support workers reported that equipment was often “‘posted-out”, with little help with effective use of equipment for a person. This may have led to further challenges for people with visual impairments, or due to a lack of training or installation by sensory support, they may not have felt confident using it. The sensory support workers also stated that training with low-vision aids can be difficult, and providing training through phone calls may not have been as effective, further hindering accessibility for people with visual impairments. In addition, without in-person meetings, many suggested that people were less open on phone calls about their struggles or queries, further impacting their sensory support care. Without an assessment of a person in their home environment, it was also very difficult to implement effective individualised rehabilitation and support strategies. 

Certification of visual impairment (CVI) was also impacted, with certifications papers being “posted out”. This suggests that the support for people with a visual impairment may have been delayed or impacted. This means that financial, emotional and physical support was therefore delayed. The results from our own unit demonstrate how dramatically CVI certification and registration dropped during the first 4/12 of COVID service restrictions [10]. An analysis of the data from the current year suggests a worrying post-COVID increase in certifications due to Age-Related Macular Degeneration (AMD) in particular. This therefore means that any sensory support has potentially been delayed further, negatively impacting these patients’ mental health and ability to adapt to sensory loss. 

People receiving treatment also expressed concerns about them losing further vision due to delays in treatment. This was a very real fear, coupled alongside the fear of COVID-19 and going out. This was especially pertinent in people with wet AMD, who were potentially more at risk due to their age but also due to the fact that their monthly treatment/treatment every 8 weeks required attendance at a clinic. Previous studies have shown that 45% of people feared losing their sight due to delayed appointments [4,8]. Our study shows that some of the reasons for this were hospital cancellations, a lack of available transport and a lack of accompaniment at appointments. A lack of accompaniment was especially problematic for elderly visually impaired users, who often had other mobility issues. This lack of accompaniment could also have impacted someone’s mental health and emotional wellbeing further should they have received a difficult or life-changing diagnosis alone.

The sensory support workers reported that care homes and day centres reduced their hours or cancelled them altogether. Reports suggest that due to a lack of ability to rehabilitate them due to restrictions and staff availability and sickness, many patients just had to go into care homes, meaning they could no longer live in their own homes and receive the appropriate support. This created a loss of independence and living skills, and many likely did not ever go home. This led to further burden and isolation for and physical detriment to a person but also a person’s family, as no respite was available. Home visits were only carried out in an emergency, and often, carers were unavailable, or appointments with them were cancelled by the recipients, causing further isolation, a lack of support and detriment to physical and mental wellbeing [8,17]. In addition, our study showed that often, different Trust areas had different approaches to sensory support, with home visits only conducted in completely necessary situations. This regional inconsistency may have been of further detriment to the visually impaired community. 

Many reported unhappiness with healthcare and social care appointments during the pandemic [9]. Our results further echo that people feared contracting COVID-19 and would not go to healthcare appointments or accept in-home support as a result [4]. Some of our cases above suggest that even those who had acute physical or mental health problems would not go to hospital for help. In fact, one very ill person simply refused to go to hospital until the sensory support worker was able to “talk them round”. With reductions in the services and space in clinics came tele-health appointments. These were not well received by people with sensory impairments, especially those who had a hearing impairment. Previous studies have shown that 21% were concerned with tele-health consultations being accessible [14]. The sensory support workers within our study were concerned that the lack of face-to-face visits was an “injustice” to their clients. While the sensory support workers were able to provide assistive devices and technology for dual sensory loss, such as sign language and video relay services, this was very difficult without face-to-face contact. Many people reported issues with accessing the appropriate primary care throughout the pandemic. They reported that patients often played down any issues they were facing and therefore did not get the correct amount of, or the appropriate, healthcare support. Despite the efforts of “bubbling” to increase social contact during the COVID-19 pandemic, this may have meant that someone only saw one other person. This could lead to further isolation for a person, overreliance on the visitor and a greater burden on the person looking after and visiting the other.

In our study, technology’s availability and acceptance were deemed to be a real issue, especially in the ageing population. Many people did not own technology or have Wi-Fi access pre-pandemic, and rural areas reported poor signal. With the increased uptake of technology over the pandemic, there were supply issues and rising costs. The financial burden of having to buy new technology (especially accessible technology) and Wi-Fi could have been a further barrier. People with children also faced challenges with online home schooling, and little support was provided on technology use. Educators with visual impairments may also have faced issues with the accessibility of the technology they were now expected to use [5]. Other working people may also have faced similar issues with accessible technology, new tele-meetings and the new “faceless” way of working [5]. 

The results from this study also reflect that people with a visual impairment could not access appropriate and accessible information on COVID-19 prevention and testing [4]. The sensory support workers reported that “bus drivers were very strict” in order to protect themselves and the public, but often, people who did not wear masks were “kicked off the bus”. As mentioned above, masks were difficult for people with a visual impairment to use, and often, people had an exemption and therefore were potentially not able to use public transport, which they often heavily relied on. Our study also mentioned extensive issues surrounding lateral flow testing, which was not accessible for people with low vision, which includes a high percentage of the elderly population. Testing was necessary for hospital appointments and surgeries and was often not completed if a sensory support or care worker was not able to be present. The small nature of the tests, the dropper and the many different elements required to complete the test were challenging for people with low vision. In addition, the poor contrast of the lines, in addition to the small test area, made interpretation incredibly difficult. 

Online shopping was also deemed to be an issue for 58% of people [5]. People with a visual impairment found it difficult to get online shopping slots, as others were now using these slots. Reports suggest that 21% of people were rationing food out of fear of going out [7], with 49% reporting getting someone else to get their shopping, as opposed to 18% pre-pandemic [7]. 

All of the barriers mentioned in our and other studies led to issues of isolation and loneliness, which can detrimentally impact physical and mental health. The results from this study echo other studies showing an increased reliance on other people [5], the negative impact of COVID-19 on isolation [5,8], its negative socio-economic impact [2] and reduced confidence [2,8]. Reports also suggest that people with a visual impairment or another disability suffered higher levels of loneliness than those without disabilities [3]. This study has also set out new information regarding the following: challenges with lateral flow testing (especially with relation to interpreting low-contrast test results); homeschooling; new technology use; and healthcare service issues. There were instances where non-face-to-face appointments led to serious adverse issues and also to a lack of “human” interaction and support when given life-changing diagnoses. This study especially highlights the importance of sensory support for people with a visual impairment and the impact of not being able to access accessible and correct support due to a lack of face-to-face assessments and appointments. This study focuses on sensory support workers’ experiences and views, which gives us new insight into the challenges faced by people with visual impairments in the pandemic. The sensory support workers continued to work closely with people with a visual impairment throughout the pandemic and saw the themes, trends and also isolated challenges faced. 

Some suggestions for moving forward in clinical practice are to assess the most accessible and best way for people to access their healthcare and sensory support. This may look different for everyone depending on their circumstances—whether by telephone, video call or face to face. Should an electronic healthcare assessment be needed, it should be completed in the most accessible way—for example, with audio for people with a visual impairment rather than video or with sign language interpretation or subtitling for people with a hearing impairment. Dual sensory loss will be more difficult in terms of accessibility, but technology and accessibility options should be made available. In addition, should another pandemic occur, it will be important to publicise the regulations in an accessible format, whether that be large print, braille or audio. Self-testing kits should also be made as accessible as possible, or provisions for help with testing should be made and continued for the duration of the pandemic. 

This study’s limitations include that we did not collect the opinions of the people with a visual impairment on the impact of COVID-19 during this study. This may have provided further insight into the issues they faced. In addition, due to issues with audio-recording in a busy and noisy room, we could not validate the transcripts with the scribe’s notes further. Due to the issues with the audio recordings, we could not include further information on the people giving the quotes. 

## 5. Conclusions

Globally, COVID-19 has impacted all of us in many varied ways. Increased barriers to social interaction, isolation and loneliness, remote working and restricted accessibility of services introduced additional challenges for people with visual impairments. Issues surrounding changes to the rules and regulations and the introduction of prevention measures further impacted people with visual impairments. Testing with lateral flow tests was incredibly difficult for people with low vision due to the size of the test kit’s components and instructions, a lack of design standardisation across the testing kits and issues associated with the test kit detection line contrast. All of these barriers, including communication issues with technology, phones and a lack of face-to-face contact, caused further isolation for this community.

## Figures and Tables

**Table 1:** Themes, codes and subcodes.

1—As a Result	2—Healthcare	3—Rules, Regulations, and Testing	4—Mental and Physical Health
Due to Covid - Familiarity loss - Home-Schooling - Online Shopping Technology Use - Availability - Understanding	Appointments - Cancelled - Patients not attending - Faceless Appointments - Lack of accompaniment General Practitioner (GP) - Difficult to access - Missing diagnoses Home Care - Lack of home care/care packages Services - Staff sickness - Closed due to COVID-19 - Reduced services	Lateral Flow Testing - Conducting tests - Getting tests - Interpretation Prevention measures - Social distancing - Mask wearing - One-way systems - Hand Sanitising - Family ‘bubbling’ Information provided - Negative	Mental Health and Isolation - Isolation - Staying at home - Anxiety - Confidence - Depression Fear of COVID-19 Public Perception

**Table 2:** Example quotes from the focus groups.

Theme	Quotes
As a result	“familiarity was gone” (Focus Group 1) “Loss of orientation which is fundamental” (Focus Group 1) “Home schooling, technology {was difficult}” (Focus Group 3) “Shopping—online and not accessible” (Focus Group 1)
Healthcare	“[General Practitioner] referral to sensory services regarding eye poking, follow up call by social worker (Sensory Services) declined a visit, due to concerns, rehab worker called again, engaged and serious support required. A result of NON face contact” (Focus Group 1) “most appointments were done by telephone (video for people needing sign language)” (Focus Group 4) “parents accepting new diagnosis for their child over the phone” (Focus Group 3) “Varied—increases interest in Zoom and Facetime—tele-conferencing, some kept going on [landline] phone” (Focus Group 4) “the amount of people who are rehabilitated had to go into care due to lack of physical services” (Focus Group 5) “people in hospital unable to be discharged home due to shortage in domiciliary care, therefore discharged to care home which resulted in decline of independence” (Focus Group 2) “There was a change in what care Sensory Support could deliver” (Focus Group 4) “offices closed. Telephone assessment: rehab services stopped by that—No Orientation and Mobility” (Focus Group 7)
Rules, regulations and testing	“didn’t know how to get tests—missed info on TV, couldn’t do a test, couldn’t read test—especially if they lived alone” (Focus Group 6) “difficulty seeing and using sanitiser/locating Personal Protective Equipment.” (Focus Group 4) “Mask wearing and impact on patients, speech to text app, linking cochlear implant to IPad” (Focus Group 6) “wearing masks added to communication difficulties.” (Focus Group 7) “1 way systems kept changing” (Focus Group 1) “people with canes find {social distancing} very difficult” (Focus Groups 5–7) “hard to maintain social distancing due to sight loss” (Focus Groups 5, 6, and 7) “couldn’t see the lateral flow, sensory worker just happened to be there and had to perform the test” (Focus Group 1) “COVID kit was virtually impossible to use if someone had low vision” (Focus Group 4) “Risk to carers of having to do tests and essentially impossible to use for patients with low vision” (Focus Group 7)
Mental and physical health	“Some people embraced tech. But in our opinion the majority rejected any offer of support” (Focus Groups 1 and 4) “people afraid to attend hospital and health deteriorates” (Focus Group 7) ‘mental health and social anxiety {impacted}” (Focus Group 5) “more dependent on others during lockdown. decline of independence. Loss of confidence” (Focus Group 2) “depression (young people do not express they are not coping)” (Focus Group 3) “so terrified of going to hospital in case they get COVID. Even people who were very seriously ill would not go to hospital (e.g., one {person} with Severe Sight Impairment)—eventually had to convince them to go and get her an ambulance” (Focus Group 7) “Older / vulnerable people still afraid to go to buildings.” (Focus Group 7) “Social workers/sensory support tried to do mostly Face 2 Face appointments but could only do these in certain cases—to deliver a replacement piece of equipment. Transport was difficult” (Focus Group 4) “struggle to get out and impact of this” (Focus Group 2) “community groups stopped or changed to Zoom, but not suiFocus Group to all” (Focus Group 4) “Overuse of technology zoom, isolated people” (Focus Group 5) “General public had poor awareness of struggles for people with a visual impairment, General public anxiety/ VI ppl getting too close to them” (Focus Group 1)

## Data Availability

The data may be accessed by contacting the corresponding author.

## References

[1] (2023). Health and Social Care, Campaign to Combat Avoidable Sight Loss in Northern Ireland. https://online.hscni.net/campaign-combat-avoidable-sight-loss-northern-ireland/#:~:text=%E2%80%9CThe%20%23EyeCareWeCare%20campaign%20also%20seeks,%2C%20community%20and%20voluntary%20sectors.%E2%80%9D.

[2] Senjam S.S. (2020). Impact of COVID-19 pandemic on people living with visual disability. Indian J. Ophthalmol.

[3] Heinze N., Hussain S.F., Castle C.L., Godier-McBard L.R., Kempapidis T., Gomes R.S. (2021). The Long-Term Impact of the COVID-19 Pandemic on Loneliness in People Living with Disability and Visual Impairment. Front Public Health.

[4] Shalaby W.S., Odayappan A., Venkatesh R., Swenor B.K., Ramulu P.Y., Robin A.L., Srinivasan K., Shukla A.G. (2021). The Impact of COVID-19 on Individuals Across the Spectrum of Visual Impairment. Am. J. Ophthalmol.

[5] Gombas J., Csakvari J. (2022). Experiences of individuals with blindness or visual impairment during the COVID-19 pandemic lockdown in Hungary. Br. J. Vis. Impair..

[6] Oviedo-Cáceres M.D.P., Arias-Pineda K.N., Yepes-Camacho M.D.R., Montoya Falla P. (2021). COVID-19 Pandemic: Experiences of People with Visual Impairment. Investig. Educ. Enferm..

[7] Royal National Institute of the Blind (2020). RNIB Responds to Inquiry on Government Response to Coronavirus. https://www.rnib.org.uk/news/inquiry-coronavirus-government-response/.

[8] Ting D.S.J., Krause S., Said D.G., Dua H.S. (2021). Psychosocial impact of COVID-19 pandemic lockdown on people living with eye diseases in the UK. Eye.

[9] Bubbico L., Bellizzi S., Ferlito S., Maniaci A., Leone Guglielmotti R., Antonelli G., Mastrangelo G., Cegolon L. (2021). The Impact of COVID-19 on Individuals with Hearing and Visual Disabilities during the First Pandemic Wave in Italy. Int. J. Environ. Res. Public Health.

[10] Jackson J., Silvestri G., Stevenson M., Sinton J., Witherow J., McCann R., Moutray T., Cushley L. (2021). COVID-19: The regional impact of COVID-19 on the certification of vision impairment in Northern Ireland. Ophthalmic Physiol. Opt..

[11] Braun V., Clarke V. (2006). Using thematic analysis in psychology. Qual. Res. Psychol..

[12] Khan H.M., Abbas K., Khan H.N. (2023). Investigating the impact of COVID-19 on individuals with visual impairment. Br. J. Vis. Impair..

[13] Lourens H. (2021). The politics of touch-based help for visually impaired persons during the COVID-19 pandemic. The COVID-19 Crisis.

[14] Active Crowd Analysis for Pandemic Risk Mitigation for Blind or Visually Impaired Persons. 2020: European Conference for Computer Vision. https://daohanlu.github.io/projects-files/active-crowd/W06P32.pdf.

[15] Cecilia R.R. (2021). COVID-19 pandemic: Threat or opportunity for blind and partially sighted museum visitors?. J. Conserv. Mus. Stud..

[16] COVID-19 and Visual Disability: Can’t Look and Now Don’t Touch. https://digitalcommons.library.umaine.edu/cgi/viewcontent.cgi?article=1078&context=c19_teach_doc.

[17] Kim H.N., Sutharson S.J. (2023). Individual differences in emotional intelligence skills of people with visual impairment and loneliness amid the COVID-19 pandemic. Br. J. Vis. Impair..

